# Near-complete elimination of mutant mtDNA by iterative or dynamic dose-controlled treatment with mtZFNs

**DOI:** 10.1093/nar/gkw676

**Published:** 2016-07-27

**Authors:** Payam A. Gammage, Edoardo Gaude, Lindsey Van Haute, Pedro Rebelo-Guiomar, Christopher B. Jackson, Joanna Rorbach, Marcin L. Pekalski, Alan J. Robinson, Marine Charpentier, Jean-Paul Concordet, Christian Frezza, Michal Minczuk

**Affiliations:** 1 MRC Mitochondrial Biology Unit, Cambridge, UK; 2 MRC Cancer Unit, Cambridge, UK; 3 GABBA, University of Porto, Portugal; 4 JDRF/Wellcome Trust DIL, Cambridge Institute for Medical Research, University of Cambridge, UK; 5 INSERM U1154, CNRS UMR 7196, Muséum National d'Histoire Naturelle, Paris, France

## Abstract

Mitochondrial diseases are frequently associated with mutations in mitochondrial DNA (mtDNA). In most cases, mutant and wild-type mtDNAs coexist, resulting in heteroplasmy. The selective elimination of mutant mtDNA, and consequent enrichment of wild-type mtDNA, can rescue pathological phenotypes in heteroplasmic cells. Use of the mitochondrially targeted zinc finger-nuclease (mtZFN) results in degradation of mutant mtDNA through site-specific DNA cleavage. Here, we describe a substantial enhancement of our previous mtZFN-based approaches to targeting mtDNA, allowing near-complete directional shifts of mtDNA heteroplasmy, either by iterative treatment or through finely controlled expression of mtZFN, which limits off-target catalysis and undesired mtDNA copy number depletion. To demonstrate the utility of this improved approach, we generated an isogenic distribution of heteroplasmic cells with variable mtDNA mutant level from the same parental source without clonal selection. Analysis of these populations demonstrated an altered metabolic signature in cells harbouring decreased levels of mutant m.8993T>G mtDNA, associated with neuropathy, ataxia, and retinitis pigmentosa (NARP). We conclude that mtZFN-based approaches offer means for mtDNA heteroplasmy manipulation in basic research, and may provide a strategy for therapeutic intervention in selected mitochondrial diseases.

## INTRODUCTION

Mitochondria are ubiquitous organelles within the eukaryotic domain, acting as a hub for numerous metabolic pathways and biochemical processes, most notably that of oxidative phosphorylation (OXPHOS). Mitochondria contain a relatively small, double-stranded, multi-copy DNA genome (mtDNA). Human mtDNA is a 16.5 kb circular molecule from which thirteen proteins and all tRNAs and rRNAs necessary for their expression are produced (1). All proteins encoded by human mtDNA are essential, hydrophobic, core subunits of the OXPHOS complexes.

Mutations in protein or RNA coding genes and regulatory elements of mtDNA often have severe biochemical consequences, resulting in mitochondrial disease. Such disorders frequently present with a heteroplasmic population of mtDNA, where typically >50% of mtDNA molecules bear a pathogenic mutation while the remainder is of the unaffected wild-type (2). Currently there are no effective treatments for mtDNA-mediated disease, and so novel approaches to therapy have been sought. One such approach, through various means, entails the selective degradation of pathogenic mtDNA in a heteroplasmic population (3,4). Such degradation results in a heteroplasmic shift, altering the proportion of mutated : wild-type mtDNA following restoration of copy number. The validity of this approach was first ascertained using mitochondrially targeted restriction endonucleases (mtREs), re-directed prokaryotic enzymes that bind and cleave specific DNA sequences with exceptional efficiency and fidelity (5–12). Unfortunately, only a very limited number of disease-causative mutations produce unique restriction sites within mtDNA, and restriction endonucleases are essentially impossible to re-engineer, either *ab initio* or *in situ*. Another reagent, also capable of such selective degradation, is the mitochondrially targeted engineered zinc finger-nuclease (mtZFN), a chimeric enzyme consisting of a Cys_2_His_2_ zinc finger protein (ZFP), conveying DNA sequence specificity, conjugated to the *Fok*I c-terminal catalytic domain, with additional mitochondrial targeting sequence (MTS) and nuclear export signal (NES) peptides ensuring exclusive mitochondrial localisation (13–15). Importantly, ZFPs can be engineered to bind virtually any DNA sequence, overcoming the targeting limitations raised in earlier work with mtREs (16,17). An alternative, engineered DNA binding technology, the *Xanthomonas*-derived transcription activator-like effector (TALE), has also been reported to produce similar effects on mtDNA heteroplasmy in comparable cybrid cell models when combined with the broadly interchangeable nucleolytic and mitochondria targeting components used in mtZFN, termed the mitochondrially targeted TALE-nuclease (mitoTALEN) (18–20).

In previous reports, we have described the construction, engineering and delivery of ZFPs or early ZFN designs to mitochondria (15,21–23). Most recently, we have reported our current heterodimeric mtZFN architecture, capable of inducing modest shifts of mtDNA heteroplasmy in both m.8993T>G point mutation and ‘common deletion’ cybrid cell models, producing a phenotypic rescue in the latter (24). In this work we describe the development of an approach to significantly enhance heteroplasmic shifts of mtDNA using mtZFN in a cybrid cell model bearing the m.8993T>G mutation; this mutation results in L217R substitution at a highly conserved residue in subunit *a* of ATP synthase, associated with neuropathy ataxia retinitis pigmentosa (NARP) syndrome (25). We compare the efficiency of our enhanced mtZFN strategy with that offered by alternative approaches to shifting mtDNA heteroplasmy with mitochondrially targeted nucleases (mtREs and TALENs). Finally, we show that our improved mtZFN approach produces physiological rescue of the m.8993T>G model, revealing a substantial metabolic shift between healthy and disease states.

## MATERIALS AND METHODS

### Plasmids and constructs

Constructs encoding mtZFN(+/−), mtTALEN(+/−) and mt-*Xma*I were as reported for the second generation mtZFN (mtZFN^2G^) in previous work, with the exception of the DNA binding domains, and cleavage domain in the case of mt*Xma*I (24). The mtZFN(+/−) and mtTALEN(+/−) constructs contain modified *Fok*I domains (ELD/KKR) that exhibit efficiency comparable to the wild-type nuclease, but with a >40-fold reduction in homodimer activity (26). These plasmids are available through the Addgene repository (https://www.addgene.org/Michal_Minczuk/). All subcloning was carried out in pcDNA3.1(−) (Invitrogen). For experiments where FACS was used, constructs bearing the HA epitope tag were cloned into pcmCherry3.1(−) between 5′ XbaI and 3′ KpnI sites; constructs bearing the FLAG epitope tag were cloned into pTracer CMV/Bsd between 5′ and 3′ PmeI sites (Life Technologies). For further detail, please see our recent methods paper (27). TALE domains were assembled according to the Unit Assembly method (28) and verified by DNA sequencing. In an attempt to encourage cleavage of mutant mtDNA and discourage cleavage of wild-type mtDNA, the TALE domains were designed such that the mutant G was in the 5′ half of the target sequence (29).

### Maintenance and transfection of mammalian cell lines

Human osteosarcoma 143B cell lineages were used in this work. Cells were cultured in Dulbecco's Modified Eagle's Medium (DMEM) containing 2 mM l-glutamine, 110 mg/L sodium pyruvate (Life Technologies) and 10% FCS (PAA Laboratories). Cells were transfected with indicated plasmids using Lipofectamine 2000 to the manufacturer's instructions. When spiking media with tetracyclines, cells were transfected in media without the drug, and this media was replaced with drug supplemented media after 5 h. After FACS at 24 h post-transfection, cells were returned to media without drug supplementation for the remainder of the experiment. In the case of stably transfected cells, DMEM as previously described, was supplemented with 1 mg/ml neomycin and applied to transfectants 24 h post-transfection. Cells were passaged for ∼18 days, and continued cultures of such cell lines were grown in DMEM as previously described, supplemented with 300 μg/ml neomycin. The m.8993T>G cybrid cells were gifted by Prof. Eric Schon.

### Cell fractionation

Fractionation experiments were performed as previously described in (30).

### Immunodetection of proteins

Localisation of proteins by immunofluorescence was carried out in fixed 143B cells as previously described (22). Images were captured using a Zeiss LSM 880 confocal microscope.

The following antibodies were used for immunofluorescence experiments in this work: mouse anti-TOM22 (Abcam, ab10436, 1:250), Alexafluor-594 anti-mouse (Molecular Probes, A11005, 1:1000), rabbit anti-TFAM (gifted by Prof. Rudolf Wiesner, 1:500), Alexafluor-405 anti-rabbit (Molecular Probes, A31553, 1:1000), rat anti-HA (Roche, 11867431001, 1:500), Alexafluor-488 anti-rat (Molecular Probes, A11006, 1:1000). Mounting medium used was either ProLong Gold Antifade Mountant (Molecular Probes), or ProLong Gold Antifade Mountant with DAPI (Molecular Probes).

For western blot analyses, ∼20 μg of extracted proteins were resolved on SDS-PAGE 4–12% bis-tris gels (Life Technologies). The following antibodies were used for western blotting in this work: mouse anti-FLAG (Sigma, F1804, 1:2000), rabbit anti-FLAG (Sigma, F7425, 1:2000), rat anti-HA (Roche, 11867431001, 1:1000), rabbit anti-Histone H4 (Abcam, ab10158, 1:5000), rabbit anti-SSB1 (kindly gifted by Prof D. Kang, 1:4000), mouse anti-TOM22 (Abcam, ab10436, 1:5000), mouse anti-GAPDH (Abcam, ab9484, 1:10 000), goat anti-rabbit HRP (Promega, W401B, 1:2000), goat anti-mouse HRP (Promega, W402B, 1:2000), goat anti-rat HRP (Santa Cruz, SC2065, 1:1000).

### FACS, mtDNA heteroplasmy and copy number analyses

Methods used to sort cells and assess mtDNA heteroplasmy and copy number are described in detail in our recent methods paper (27). All FACS was carried out at the NIHR BRC Cell Phenotyping Hub, Cambridge, UK.

### Ultra-deep mtDNA amplicon re-sequencing

A single 682 bp amplicon, corresponding to nt.8657-9339 of human mtDNA encompassing the mtZFN target site and ∼340 bp upstream and downstream regions of the predicted breakpoint, was produced by PCR using KOD polymerase (Toyobo) according to the manufacturer's instructions with a previously published primer pair (24,27). This PCR amplicon was then subjected to Illumina Nextera sample processing and 300-cycle paired-end sequencing using an Illumina MiSeq instrument.

Paired-end sequences were aligned to the rCRS mitochondrial genome (NC_012920) using BWA (MEM mode) (31) and sorted using Picard tools. Read qualities and alignments were evaluated using Mpileup of SAM tools (32). Reads were discarded if they had a mapping phred score of less than 30, and bases were ignored if they had a base quality phred score of less than 30. All reads were included, without subsampling. To detect variants in mtDNA, the output of Mpileup was analysed using VarScan (v2.3) (33) in SNP and indel modes, with a minimum variant frequency of 0.05%, and minimum depth of 1000 reads. Variants with a frequency of <0.5% were considered likely non-significant, as the distribution of each of the four bases suggested this was the limit of sequencing accuracy.

### Oxygen consumption rate measurements

Oxygen consumption rate (OCR) was measured using the real time flux analyser XF-24e (Seahorse Bioscience). For these experiments 6 × 10^4^ cells were treated with 1 μM Oligomycin, 2 μM Carbonyl cyanide-*p*-trifluoromethoxyphenylhydrazone (FCCP), 1 μM Rotenone and 1 μM Antimycin A (all purchased from Sigma-Aldrich). At the end of the run cells were lysed using RIPA buffer (25 mM Tris–HCl pH 7.6, 150 mM NaCl, 1% NP-40, 1% sodium deoxycholate, 0.1% SDS). Protein content for each well was measured using BCA kit (Pierce) according to the manufacturer's instructions. OCR was normalised to total protein content. The extent of ATP-linked mitochondrial respiration was obtained by calculating the ratio between OCR in the presence or absence of Oligomycin. Data were obtained from 3 independent experiments and presented as mean ± SEM. Statistical analysis was carried out with Prism Graphpad software by applying one-way ANOVA and Tukey post-hoc test (FDR = 0.05).

### Liquid chromatography coupled to mass spectrometry (LC–MS) metabolomic analysis

For metabolomic studies, 1 × 10^5^ cells were plated onto a 12-well plate and cultured in standard conditions for 24 h. Media was then replenished and after 24 h intracellular metabolites were extracted as previously described (34). LC–MS analysis was performed on a QExactive Orbitrap mass spectrometer coupled to a U3000 LC system (Thermo). The liquid chromatography system was fitted with a Sequant Zic-pHilic (150 mm × 2.1 mm, internal diameter 3.5 μm) with guard column (20 mm × 2.1 mm, internal diameter 3.5 μm) from HiChrom, Reading, UK. The mobile phase was composed of 20 mM ammonium carbonate and 0.1% ammonium hydroxide in water (solvent A), and acetonitrile (solvent B). The flow rate was set at 180 μl x min^−1^ with the following gradient: 0 min 80% B, 28 min 20% B, 29 min 80% B, 45 min 80% B. The mass spectrometer was operated in full MS and polarity switching mode. Samples were randomized in order to avoid machine drift and blinded to the operator. The acquired spectra were analysed using XCalibur Qual Browser and XCalibur Quan Browser softwares (Thermo Scientific) by referencing to an internal library of compounds. Integrated metabolites were normalised on total metabolite intensity per sample and statistically significant metabolites were obtained by applying ANOVA and Tukey's post-hoc test between samples (*n* = 4). Statistical analysis of metabolomic data was carried out with R software (FDR = 0.05).

### Energy charge state analysis

Energy charge state analysis for cybrids cells was calculated using values for adenosine phosphate species as detected by LC-MS, expressed as (ATP + $\frac{1}{2}$ ADP)/(AMP + ADP + ATP), by the established method (35). The energy charge for most cell types typically ranges from 0.8 to 0.95.

## RESULTS

### Modifying mtDNA heteroplasmy upon short-term expression of mtZFNs

**Figure 1.**
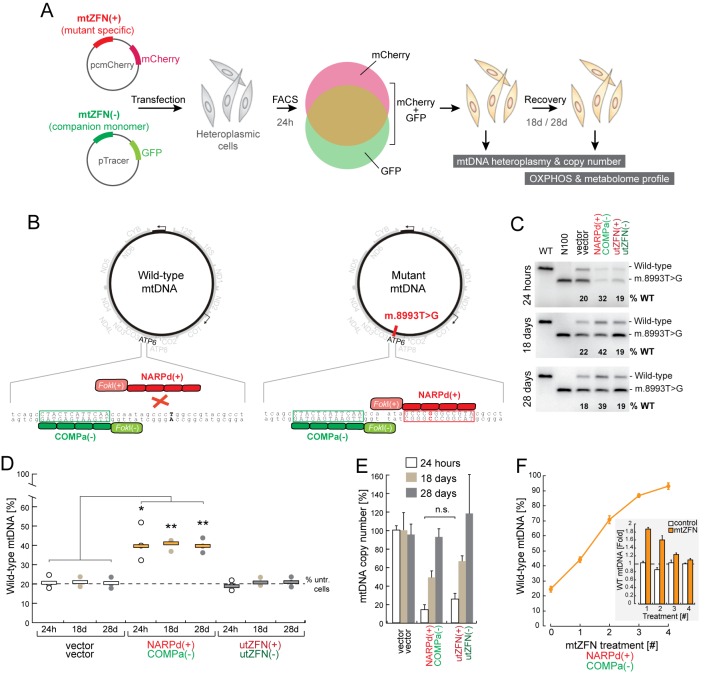
Strategy for targeting the m.8993T>G NARP mutation using mtZFN and analysis of heteroplasmy and copy number upon short-term expression of mtZFNs. (**A**) Schematic of the general workflow for experiments that involve transient transfection of heteroplasmic cells with plasmids co-expressing mtZFN monomers and fluorescent marker proteins, FACS-based selection of cells expressing both mtZFN monomers and phenotypic evaluation of mtZFN-treated cells. The technical details and plasmids are as described in (27). (**B**) Detailed schematic of strategy for selective degradation of m.8993T>G mtDNA using mtZFN. Conventional dimeric, engineered mtZFN are directed to sequence adjacent to (COMPa, green) or including (NARPd, red) the mutated base position. Both monomers should bind the substrate only when the indicated nucleotide is mutated and not to the wild-type sequence. DNA double strand breaks should only be introduced into the mutant mtDNA molecule, leading to a shift in heteroplasmy. (**C**) Last cycle hot PCR restriction fragment length polymorphism (RFLP) analysis of mtDNA from FACS-enriched cells transiently expressing indicated mtZFN construct pairs. 143B and N100 are included as wild-type and 100% mutant digestion controls, respectively. (**D**) Quantification of RFLP heteroplasmy data from several biological replicates of FACS-enriched cells transiently transfected with mtZFN or control vectors. Data presented are from measurements made at 24 h, 18 days and 28 days post-transfection, as indicated. Statistical analyses were carried out using a two-tailed Student's *t*-test, *P* = 0.027 (24h); *P* = 0.00167 (18 days); *P* = 0.00146 (28 days); *n* = 3. ‘% untr. cells" indicates the baseline heteroplasmy of the N80 cell line used in these experiments. (**E**) Analysis of mtDNA copy number, performed by qPCR in quadruplicate, from samples tested in (C). Error bars = 1 S.D. (**F**) Wild-type mtDNA heteroplasmy shifts upon iterative expression and recovery cycles of mtZFN pairing NARPd(+)/COMPa(−). Inset; quantification of fold-changes in mtDNA heteroplasmy for mtZFNs and controls for each iteration of transfection/FACS/recovery. Measurements of heteroplasmy presented were taken 28 days post-transfection. ‘% untr. cels’ indicates the baseline heteroplasmy of the N80 cell line used in these experiments.

We addressed the feasibility of targeting pathogenic mtDNA mutations using short-term, high expression of mtZFN. To this end, a previously published mtZFN pairing shown to be specific to the m.8993T>G mutation, NARPd(+) and COMPa(−) (21,24), were cloned into vectors that co-express fluorescent marker proteins (mCherry for mtZFN(+), GFP for mtZFN(−)) enabling fluorescence activated cell sorting (FACS) of transiently transfected cells (Figure 1A and B). Such a strategy allows for much greater temporal resolution of nuclease activity in mitochondria than transfection/selection approaches, with increased confidence in the homogeneity of samples analysed (27). In these experiments, we included additional control mtZFNs that are delivered to mitochondria but contain ZFP domains **u**n**t**argeted to mtDNA (utZFN(+)/(−)), serving as an indicator of non-specific or off-target activity. We transfected constructs and empty vectors into 143B cybrid cells bearing ∼80% m.8993T>G mtDNA (N80), which were subjected to FACS at 24 h post-transfection, enriching the population of cells expressing the transfected constructs. After FACS, we harvested a subset of cells and seeded the remaining transfectants for continued culture, collecting these cells at 18 days and 28 days post-transfection for mtDNA heteroplasmy and copy number analyses (Figure 1A). The m.8993T>G mutation produces a unique *Sma*I/*Xma*I restriction site, and heteroplasmy of sorted cells was determined using a last cycle hot polymerase chain reaction (LCH-PCR) assay, followed by *Sma*I restriction fragment length polymorphism (RFLP), as previously described (24,27) (Figure 1C). This assay indicated that the NARPd(+)/COMPa(−) mtZFN pairing was capable of producing a modest mtDNA heteroplasmy shift, from ∼22% to ∼40% wild-type mtDNA, as measured at 24 h, which remained stable 18 days and 28 days post-transfection (Figure 1D). This result is broadly in line with our previously published data on this optimized mtZFN pairing, obtained from experiments examining the effects of long-term mtZFN expression facilitated by antibiotic selection (24). However, copy number measurements from these cells indicated a significant depletion of total mtDNA levels, to ∼15% of control, at 24 h post transfection, recovering to ∼50% of control by 18 days post-transfection and achieving parity with control by 28 days post-transfection (Figure 1E). Interestingly, the utZFN(+)/(−) pairing did not induce a shift in heteroplasmy, though still produced a large depletion of mtDNA copy number, to ∼25% of control at 24 h post-transfection, increasing to ∼65% of control at 18 days post-transfection and recovering to parity with control by 28 days post-transfection (Figure 1D and E). To ensure that the RFLP assay used was not confounded by repair of mtDNA molecules through non-homologous end joining (NHEJ), which might result in abolition of the *Sma*I restriction site, we performed ultra-deep re-sequencing of the PCR amplicon used for RFLP. This analysis did not detect any unexpected sequence variants or variation in insertion or deletion formation in mtZFN treated cells compared with controls, confirming the fidelity of the RFLP assay (Supplementary Figure S1). That repaired molecules were undetectable at such a sequencing depth strongly suggests either a non-existent or extremely inefficient NHEJ double-strand break repair pathway in mammalian mitochondria. These data, taken together, suggest that although short-term, high-dosage expression of mtZFNs produce significant shifts in mtDNA heteroplasmy, substantial off-target effects were occurring in these conditions, leading to undesired mtDNA copy number depletion.

### Iterative treatment of m.8993T>G with mtZFN results in a near-complete heteroplasmy shift

Next, we set out to determine the effect of repeated mtZFN treatments on mtDNA heteroplasmy shifting. To this end, we performed several sequential rounds of transfection and recovery using the m.8993T>G heteroplasmic cells and NARPd(+)/COMPa(−) mtZFNs. Briefly, this consisted of transfection and selection of transfectants by FACS at 24 h, followed by a 28 day recovery period to allow mtDNA copy number repletion, at which point heteroplasmy was measured and cells were re-transfected. Beginning with N80 cells, using four iterative cycles of transfection and recovery, this approach was capable of producing a cumulative shift in heteroplasmy from ∼80% to ∼7% m.8993T>G mtDNA (Figure 1F). These results demonstrated that sequential, short-term mtZFN treatments, despite the significant off-target activity observed (Figure 1E), could achieve near-complete elimination of mtDNA mutations.

### A FACS approach to modify dosage of mtZFN

**Figure 2.**
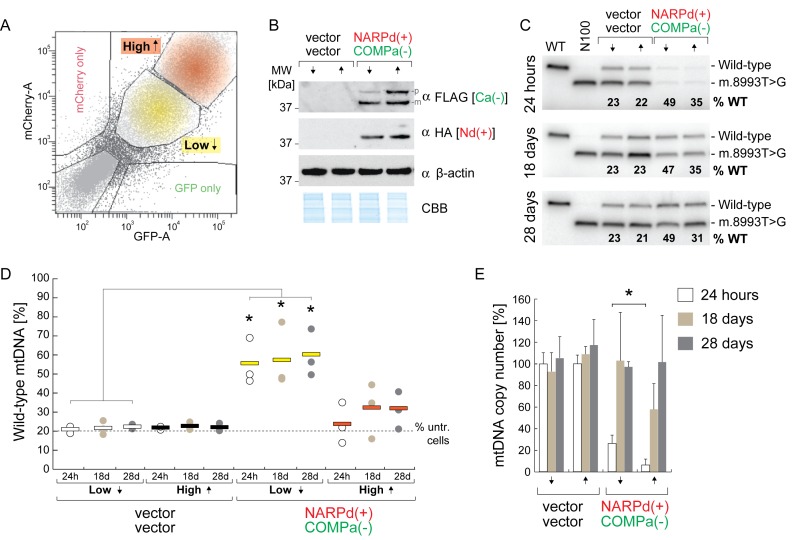
Dosage of mtZFN substantially alters the efficiency of heteroplasmy shifting and mtDNA copy number depletion/repletion profiles. (**A**) Representative dot plot indicating a typical FACS gating strategy used to separate ‘high’ and ‘low’ mtZFN-expressing transfectants. (**B**) Western blot analysis of total cellular proteins from FACS-enriched samples at 24 h post-transfection by SDS-PAGE, probing for mtZFN expression with antibodies to the HA (NARPd (Nd)) or FLAG (COMPa (Ca)) epitopes. β-actin and a section of Coomassie stained gel (CBB) are shown as loading controls. p, precursor isoform; m, mature isoform. (**C**) Last cycle hot PCR RFLP of mtDNA from cells transiently expressing ‘high’ or ‘low’ quantities of mtZFN or control vectors. (**D**) Quantification of collated heteroplasmy data from several biological replicates of transient mtZFN or control vector expression. Data presented are from measurements made at 24 h, 18 days and 28 days post-transfection, as indicated. Statistical analyses were carried out using a two-tailed Student's *t*-test, *P* = 0.044 (‘low’ 24 h); *P* = 0.032 (‘low’ 18 days); *P* = 0.011 (‘low’ 28 days); *n* = 3. ‘% untr. cells’ indicates the baseline heteroplasmy of the N80 cell line used in these experiments. (**E**) Analysis of mtDNA copy number, performed by qPCR in quadruplicate, from samples tested in D. Statistical analyses were carried out using a two-tailed Student's *t*-test, *P* = 0.036 (mtZFN, 24 h). Error bars = 1 S.D.

Having identified excessive depletion of mtDNA copy number as potentially obstructive to our purpose, we intended to test whether a lower concentration of mtZFNs in mitochondria could minimise this effect. To this end, we modified our previous FACS-based approach, isolating heteroplasmic m.8993T>G cells transiently expressing variable quantities of mtZFN by altering the gating strategy employed. We were able to extract two distinct populations of transiently transfected cells, termed ‘low’ and ‘high’ (Figure 2A), which, we hypothesised, would express varied quantities of the transfected constructs in line with detected fluorescence. N80 cells were transiently transfected with NARPd(+)/COMPa(−) mtZFN constructs or empty vectors, and were subjected to FACS after 24 h with transfectants separated into ‘high’ and ‘low’ populations. Western blotting revealed differences in mtZFN protein expression levels between these two populations (Figure 2B). At 24 h post-transfection, in the ‘low’ expressing mtZFN condition, we observed an increase in effectiveness of mtDNA heteroplasmy shifting, from ∼22% to ∼55% wild-type mtDNA (Figure 2D), as compared to the total transfectant pool, which shifted from ∼22% to ∼40% wild-type mtDNA (Figure 1D). We also observed an improved rate of mtDNA copy number recovery in the ‘low’ expressing population, where parity with control was achieved at 18 days post-transfection (Figure 2E), as compared to the total transfectant pool, which reached parity with control at 28 days post-transfection (Figure 1E). Heteroplasmic shifts in these cells remained stable at 18 and 28 days post-transfection (Figure 2D). However, heteroplasmic shifts observed in the ‘high’ expressing mtZFN condition were substantially diminished, to levels that were no longer considered statistically significant (Figure 2D). We also observed greater mtDNA copy number depletion in the ‘high’ expressing conditions, to ∼7% of control, at 24 h post-transfection, as compared to the ‘low’ expressing transfectant population, which depleted mtDNA copy number to ∼24% of control (Figure 2E). These data suggested that an optimised ‘dose’ of mtZFNs can enhance mtDNA heteroplasmy shifts and minimise mtDNA copy number depletion.

### A hammerhead ribozyme-based approach for fine control of mtZFN expression

**Figure 3.**
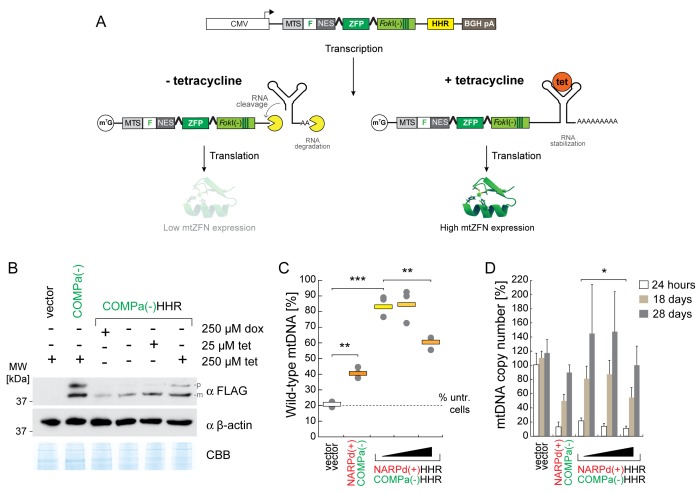
Dynamic control of mtZFN expression using a tetracycline-sensitive 3′ hammerhead ribozyme (HHR) reveals dosage-dependent effects on heteroplasmy and mtDNA copy number depletion/repletion profiles. (**A**) Schematic of mtZFN transgene with the hammerhead ribozyme (HHR) incorporated. When transcribed, mRNA encoding mtZFN is constitutively degraded following HHR cleavage, resulting in substantially lower quantities of translated protein. However, in the presence of tetracycline the HHR element is stabilized, inhibiting catalysis and producing larger quantities of translated protein. CMV, cytomegalovirus promoter, MTS, mitochondrial targeting sequence, F, FLAG epitope tag, NES, nuclear export signal, BGH pA, bovine growth hormone polyadenylation signal. (**B**) Western blot analysis of total cell lysates from FACS-enriched samples at 24 h post-transfection, probing for expression of COMPa(−) mtZFN or COMPa(-)HHR mtZFN (FLAG) in the indicated concentrations of tetracycline (tet) or doxycycline (dox) in the medium. β-actin and a section of Coomassie stained gel (CBB) are shown as loading controls. p, precursor isoform; m, mature isoform. (**C**) Quantification of collated heteroplasmy data from several biological replicates of transient mtZFN expression. Measurements made at 28 days post-transfection. Filled black triangle indicates spectrum of tetracycline concentrations (0, 25, 250 μM). *n* = 3, statistical analyses were undertaken using a two-tailed Student's *t*-test, *P* = 0.0015 (vector/mtZFN), *P* = 0.0002 (vector/mtZFN-HHR, 0 μM), *P* = 0.009 (mtZFN-HHR, 0 μM/mtZFN-HHR, 250 μM). ‘% untr. cells’ indicates the baseline heteroplasmy of the N80 cell line used in these experiments. (**D**) Analysis of mtDNA copy number, performed by qPCR in quadruplicate, from samples tested in C. Statistical analyses were undertaken using a two-tailed Student's *t*-test, *P* = 0.047. Error bars = 1 SD.

As alterations of mtZFN concentration in mitochondria were observed to produce significantly improved shifts in mtDNA heteroplasmy, with reduced deleterious effects on mtDNA copy number, we sought a method to exercise greater control over protein expression levels. Recent advances in RNA chemistry have yielded an array of engineered hammerhead ribozymes (HHRs), modified bacterial ribozymes that constitutively cleave the RNA species within which they are contained. In the case of a eukaryotic mRNA, if placed upstream of the poly(A) signal, HHR-mediated cleavage produces a 3′ end that is susceptible to degradation, greatly reducing protein expression from this mRNA (36). This catalytic RNA element has recently been combined with a RNA aptamer, specific to the antibiotic drug tetracycline. Binding of tetracycline rigidifies the RNA structure and inhibits catalysis by the HHR (37). We incorporated the 3′K19 version of this HHR element into our mtZFN(+)/(−) backbone, between the mtZFN ORF stop codon and the bovine growth hormone (BGH) poly(A) site (Figure 3A). Importantly, any such system of regulation applied equally to a bipartite catalytic mechanism is likely to entail significant cooperative effects on the rate of nucleolytic cleavage, enabling a broad dynamic range of catalytic control.

To assess the dynamic range of mtZFN dosage control exerted by 3′K19 HHR, we transfected wild-type 143B cells with a single mtZFN monomer (COMPa(-)), either with or without the 3′ K19 HHR element. COMPa(−)HHR transfected cells were grown in media supplemented with various concentrations of tetracycline or doxycycline (Figure 3B). We enriched transfectants by FACS at 24 h post-transfection, sorting GFP positive cells only. Western blot analysis of COMPa(−) protein levels revealed a substantial change in expression of the protein between various drug concentrations, appearing broadly in line with the previously published data where *cis*-catalysis of mRNA by the 3′K19 HHR reduced expression of the transgene by ∼90% (37). (Figure 3B). Expression of COMPa(−)HHR was not altered in the presence of 250 μM doxycycline, as compared to COMPa(−)HHR in the presence of 0 μM tetracycline, demonstrating the specificity of the RNA aptamer for tetracycline only. In these conditions, expression of mtZFN was not detectable at 18 days or 28 days post-transfection (Supplementary Figure S2).

Next, we transiently transfected N80 cells with the NARPd(+)HHR/COMPa(-)HHR constructs, and grew them for 24 h in media supplemented with different concentrations of tetracycline prior to FACS. Analysis of m.8993T>G heteroplasmy at 24 h indicated moderately enhanced shifts in cells transfected with NARPd(+)HHR/COMPa(−)HHR constructs as compared to mtZFN not containing 3′K19 HHR (Supplementary Figure S3A). However, continued culture of m.8993T>G cybrid cells transfected with NARPd(+)HHR/COMPa(−)HHR up to 28 days post-transfection revealed much more pronounced heteroplasmy shifts towards wild-type mtDNA (Figure 3C and Supplementary Figure S3B). Importantly, in conditions exhibiting the lowest mtZFN expression levels (tetracycline-free and 25 μM), we observed a near-complete wild-type heteroplasmy rescue, from ∼20% to ∼ 90% wild-type mtDNA at 18 days post-transfection (Supplementary Figure S3B), which was stable at 28 days (Figure 3C). Treatment with NARPd(+)HHR/COMPa(−)HHR mtZFN constructs without tetracycline was accompanied by a less-pronounced mtDNA copy number depletion at 24 h post-transfection (21% of control) as compared to cells transfected with the same constructs exposed to 250 μM tetracycline (12% of control) (Figure 3D). A concurrent decrease in heteroplasmy shifting efficiency was also observed with higher expression levels of HHR-controlled mtZFNs, when comparing lower expressing (tetracycline-free or 25 μM) and higher expressing (250 μM) conditions (Figure 3C). Similarly, the rate of mtDNA copy number repletion was improved for HHR-controlled mtZFN constructs exposed to 25 μM or no tetracycline, reaching parity with control by 18 days post-transfection (Figure 3D). Additionally, we transiently transfected N80 cells with utZFN(+/−)HHR constructs in the absence of tetracycline, observing that decreased levels of non-specific nuclease expression reduce copy number depletion by >100% at 24 h post-transfection, with a substantially improved mtDNA repopulation profile, regaining parity with control at 18 days (Supplementary Figure S4). These data demonstrated that manipulating dosage of mtZFNs, through the incorporation of an engineered hammerhead ribozyme, can improve degradation of pathogenic mutations in heteroplasmic mtDNA populations by more than 2-fold beyond uncontrolled, high dosages of mtZFNs through mitigation of off-target catalytic events.

### Comparing the mtDNA heteroplasmy shifting efficiency of mtZFNs with alternative mitochondrially targeted nucleases

Next, we compared the efficiency of shifts in m.8993T>G heteroplasmy produced by HHR-controlled mtZFNs with those of a mtRE. To this end, we reproduced the HHR dose-controlled experiments presented using a mtRE, *Xma*I (mt-*Xma*I) (Supplementary Figure S5A), which binds and specifically cleaves the unique site produced in mtDNA by the m.8993T>G mutation (5′ CCCGGG ’3) at very high efficiency (10). N80 cells were transfected with mt-*Xma*I, mt-*Xma*IHHR or control vector, grown in media supplemented with various quantities of tetracycline and subjected to FACS at 24 h, gating the mCherry positive population only. Western blot analysis of these transfectants demonstrated tetracycline dose-dependent expression of mt-*Xma*I (Supplementary Figure S5B), comparable to that seen for mtZFN (Figure 3B). Under both HHR-controlled and unrestrained, CMV-driven expression conditions, mt-*Xma*I produced a shift in heteroplasmy from 20% to ∼86% wild-type mtDNA at 28 days post-transfection (Supplementary Figure S5C and D). This shift was accompanied by a significant depletion of mtDNA copy number in all expression conditions at 24 h, which regained parity with control at 18 days post-transfection (Supplementary Figure S5E). Of interest, lower expression conditions, particularly the tetracycline-free mt-*Xma*IHHR condition, appeared to shift heteroplasmy to a lesser extent at 24 h (Supplementary Figure S5F), displaying concomitantly less mtDNA copy number depletion, though heteroplasmic shifts are essentially identical by 28 days post-transfection, suggesting that, unlike for mtZFN, limiting the concentration of mt-*Xma*I is detrimental to heteroplasmy shifting activity.

We also attempted experiments using a mitochondrially targeted TALE nuclease (mtTALEN) architecture of our own design (Supplementary Figure S6A), essentially identical to the mitoTALEN design that has been reported previously (18,19). Our heterodimeric mtTALENs were efficiently delivered to mitochondria, where they co-localised with core mitochondrial nucleoid component TFAM (Supplementary Figure S6B–D). We tested four distinct TALE pairs targeted to m.8993T>G and control constructs that contained TALE domains non-specific to mtDNA (utTALEN) (Supplementary Figure S7A). None were able to shift heteroplasmy to a statistically significant extent upon high dose short-term expression, though all pairs depleted mtDNA copy number (Supplementary Figure S7B–D). The effect on mtDNA copy number indicates that mtTALENs are imported into the mitochondrial matrix, consistent with the cellular localisation studies (Supplementary Figure S6).

To investigate whether dosage dependent effects might be governing the efficiency of these constructs, we took the best mtTALEN pairing (which appeared to shift heteroplasmy in a single biological replicate) and conducted FACS-gated ‘high’ and ‘low’ dosage control experiments (Supplementary Figure S8A). These experiments demonstrated no differences in heteroplasmy between high and low expressing populations of this optimal mtTALEN pairing (Supplementary Figure S8B,C), though a significant increase in mtDNA copy number depletion was observed upon high expression of mtTALEN (Supplementary Figure S8D). The results of these experiments demonstrate that, through limited expression strategies, mtZFN are capable of shifting m.8993T>G heteroplasmy to a degree indistinguishable from a restriction endonuclease, and that mtTALEN are unable to effectively target m.8993T>G.

### Rescue of mtDNA m.8993T>G-associated mitochondrial defect by mtZFNs is associated with metabolic rewiring

**Figure 4.**
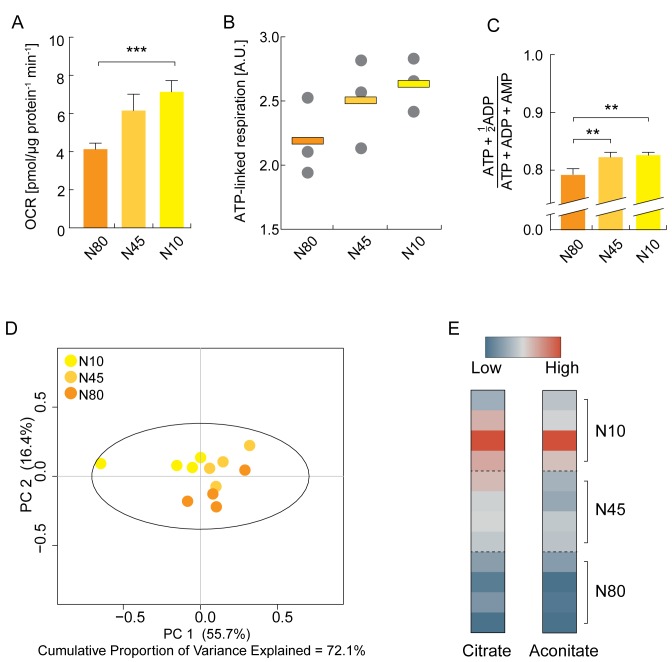
Treatment with mtZFN rescues the mitochondrial defect associated with m.8993T>G and is accompanied by rewiring of cellular metabolism. (**A**) Oxygen consumption rates (OCR) in empty vector-transfected cybrid cells harbouring 80% of m.8993T>G (N80) and cells of the same origin transfected with NARPd(+)-HHR/COMPa(−)-HHR with either 25 μM (N10) or 250 μM (N45) tetracycline present in the culture medium. These cell lines were generated concurrently within a single experiment. Statistical analysis was undertaken using a two-tailed Student's *t*-test (*P* = 0.0001865, *n* = 7). (**B**) ATP-linked mitochondrial respiration in N80, N45 and N10 cells, calculated as the ratio of OCR in the presence or absence of oligomycin. (**C**) Energy charge state analysis of N80, N45 and N10 cells, calculated using values for adenosine phosphate species detection by LC-MS by the established method (35). (**D**) Principal Component Analysis (PCA) of intracellular metabolites from N80, N45 and N10 cells as measured by LC-MS-based metabolomics. Score plot of principal component 1 and 2, explaining 55.7% and 16.1% of total variance, respectively, is shown. (**E**) Heatmap representation of intracellular levels of citrate and aconitate in N80, N45 and N10 cells, as measured by LC–MS. Of note, citrate abundance was found to be significantly different between N80 and N45, as well as between N80 and N10, while aconitate was significantly upregulated in N10 compared to N80 cells (Supplementary Figure S8).

Next we set out to determine whether mtZFN-mediated shifts in heteroplasmy of m.8993T>G mutant mtDNA were associated with rescue of mitochondrial respiratory function and changes in metabolic profile. The following cell lines were assessed for physiological changes associated with their altered heteroplasmic states: control vector-transfected N80 cells and N80-derived cells obtained following treatment with NARPd(+)HHR/ COMPa(−)HHR in 25 μM (10% m.8993T>G mtDNA; N10) and in 250 μM tetracycline concentrations (45% m.8993T>G mtDNA; N45). Oxygen consumption increased in both N45 and N10 compared to N80 (Figure 4A). Furthermore, genetic correction of m.8993T>G mtDNA increased both proportion of mitochondrial respiration coupled to ATP production and adenylate energy charge state (Figure 4B,C), indicating a functional rescue of mitochondrial respiration following heteroplasmy shift.

We then investigated whether heteroplasmy shift of m.8993T>G mtDNA is associated with changes in cellular metabolism. To this end we performed Liquid-Chromatography coupled to Mass Spectrometry (LC-MS)-based metabolomic analyses of N80, N45 and N10 cells. Principal component analysis based on a matrix of 70 metabolites clearly separated the three cell lineages based on their heteroplasmy level (Figure 4D), indicating that substantial metabolic changes are associated with genetic correction of m.8993T>G mtDNA. Univariate analysis of individual metabolites (Supplementary Figure S9) demonstrated that citrate and aconitate, two key mitochondrial metabolites, were among the most increased metabolites in both N45 and N10 compared to N80 (Figure 4E and Supplementary Figure S9). These data suggested that the defect of mitochondrial function in cells bearing high loads of m.8993T>G mtDNA can be corrected by mtZFNs.

## DISCUSSION

In this work, we strove to develop improved approaches to haplotype-specific mtDNA heteroplasmy shifting using mtZFNs. We undertook experiments with mtZFNs targeted to the m.8993T>G mutation, causative of NARP syndrome, using a well-investigated cybrid cell model. Through use of a FACS-based method for selecting populations of transiently transfected cells (Figure 1A), we observed that mtZFNs expressed at a high level (i.e. CMV-driven expression) produce considerable depletions of mtDNA copy number (Figure 1E), regardless of their effect on mtDNA heteroplasmy (Figure 1C and D). Theoretically, if the mtZFN pair were to be perfectly specific to m.8993T>G mtDNA, and exclusively cleave the mutant haplotype, it would be expected that, in N80 cells, mtDNA copy number would be depleted to 20% of control, at its lowest point, with a shift to 100% wild-type mtDNA achieved upon recovery of copy number. Therefore, it was hypothesised that substantial off-target effects are likely to be limiting heteroplasmy-shifting efficiency. Despite the significant mtDNA depletions observed in m.8993T>G-specific mtZFN treatment, we demonstrated that iterative cycles of transfection and recovery allowed stepwise increases in wild-type mtDNA heteroplasmy, achieving a cumulative shift from 20% to 93% wild-type mtDNA after four cycles of treatment (Figure 1F).

To test the hypothesis of non-specific nuclease-DNA interactions limiting the efficiency of heteroplasmy shifting, we produced a system to compare conditions of higher and lower mtZFN expression. The rationale for this was that lower mitochondrial concentrations of a nuclease would produce proportionately fewer off-target cleavage events, potentially enhancing heteroplasmy shifts. By isolating ‘high’ and ‘low’ expressing m.8993T>G cybrid cells from the same transfection using FACS (Figure 2A), we were able to produce cell populations with a modest alteration in mtZFN expression levels (Figure 2B). The proportions of pre-import ‘precursor’ and post-import ‘mature’ isoforms of mtZFN were altered depending on expression level, with ‘high’ cell populations demonstrating an increased quantity of the precursor isoform, suggesting temporary saturation of the mitochondrial protein import apparatus. The ‘low’ expression levels were associated with less-severe depletions of mtDNA copy number and a concomitant improvement in heteroplasmy shifting (Figure 2C–E), enhancing the efficiency of the treatment by ∼100% when compared with experiments where the entire transfectant population was sorted and analysed *en masse* (Figure 1). Cells from the ‘high’ expression condition, transfected with the same mtZFNs, demonstrated an abrogated capacity to shift heteroplasmy, accompanied by a noticeable increase in severity of mtDNA copy number depletion (Figure 2C and D).

With these data suggesting that relatively small changes in mtZFN expression levels could drastically alter the efficiency of their DNA-specific activity, we set out to generate a universal platform allowing dynamic control of transient mtZFN expression. To achieve this we incorporated a self-cleaving 3′ hammerhead ribozyme (HHR) element, engineered to include a well-characterised tetracycline-specific RNA aptamer as a means of disinhibition (37), providing the basis for dynamic, dose-controlled expression of mtZFNs within a transient expression system (Figure 3A). The constitutive self-cleaving activity of the HHR catalytic RNA greatly attenuated expression of the mtZFN monomers, and addition of tetracycline to the culture medium inhibited RNA cleavage, permitting expression of mtZFNs to near CMV-driven levels (Figure 3B). The reduced mtZFN expression levels observed in tetracycline-free or 25 μM tetracycline conditions produced heteroplasmy shifts resulting in >4-fold greater quantities of wild-type mtDNA compared with mock transfected cells (Figure 3C), and >2-fold greater quantities when compared with experiments assessing the entire population of CMV-driven mtZFN transfectants (Figure 1C–E). The severity of mtDNA copy number depletion in these cells was also greatly decreased (Figure 3D). Further, measurements of copy number depletion in cells expressing various amounts of utZFN(+/−) demonstrated that low levels of expression improve the copy number depletion/repletion profile (Supplementary Figure S4). These data confirm the relationship between over-dosage of mtZFNs and loss of heteroplasmy shifting efficacy, as a function of unintended mtDNA copy number depletion through off-target effects. The precise nature of off-target catalysis observed in these studies, whether at the mutation site, at other similar sites or entirely non-specific is uncertain, though experiments with utZFN and utTALEN suggest that entirely non-specific nucleolytic effects form a substantial component of this milieu.

The comparisons made between mtZFN technology and the better-investigated mitochondrially targeted restriction endonucleases (5–12), in this case mt-*Xma*I, clearly indicate that the capacity of *Xma*I to reject an incorrect substrate and avoid off-target activity-mediated mtDNA copy number depletion is considerably greater than that of mtZFNs (Supplementary Figure S5). This result is not unexpected, given the published *in vitro* data on *Xma*I, which indicates ∼1000-fold greater affinity for canonical over non-cognate sites, defined as a sequence where 1 of 6 nucleotides is altered (38), as is the case for m.8993T>G. However, for mtZFN expression limitation strategies to be capable of achieving a shift in heteroplasmy of identical magnitude to that produced by a mtRE is encouraging, particularly with regard to future applications of this technology. Our attempts to shift m.8993T>G heteroplasmy with a TALE-based design were unsuccessful, as it appears that this site in mtDNA is not easily targeted by this technology. This lack of efficacy is likely due to the inability of TALE assemblies to act with single-nucleotide specificity at the target site, resulting in degradation of both wild-type and mutant mtDNA molecules at a similar rate. Of note, the majority of previously published mitoTALENs have exploited a key TALE binding constraint to their advantage, the necessity for a thymidine base at position 0 of a DNA target site. Without a T at position 0, binding of TALE domains can be greatly impeded (39). As such, targeting heteroplasmic mtDNA point mutations producing N>T or N>A substitutions using a TALE-based architecture can be highly efficacious. However, such a strategy is not available in the case of m.8993T>G. Furthermore, the sequence context in which a mutation is present can also determine the effectiveness of targeting using TALE-based systems. Specifically, the closer the mutated base is to the N-terminal TALE binding module, the more likely it is that the TALE will be able to discriminate with single nucleotide specificity. As a T at position 0 is highly preferred, the sequence context of m.8993T>G does not permit placement of the mutation site especially close to the N-terminal portion of the TALE module, and this is a likely reason for the lack of heteroplasmy shifting activity observed. We can also speculate as to the accessibility of this mtDNA target site by mtTALENs, which may be compromised by m^5^C methylation, or other epigenetic modifications present in the target sequence (40–43). However, our data still suggest the importance of dosage control for these constructs, as we have shown that a TALEN not specific to mitochondrial DNA sequence, expressed at a high level in mitochondria, can produce substantial mtDNA copy number depletion (Supplementary Figures S7 and S8).

Finally, to confirm that the changes in m.8993T>G mutation load are associated with altered mitochondrial function, we performed a comprehensive metabolic characterisation of cells with different, but stable levels of heteroplasmy. We found that correction of m.8993T>G mutation is associated not only with increased basal mitochondrial respiration, but also with greater coupling of mitochondrial oxygen consumption to ATP production and improvements in energy charge state (Figure 4A–C). These alterations of mitochondrial function presaged important consequences for cellular metabolism, and our metabolomic analyses pointed to a comprehensive rewiring of metabolism upon correction of the m.8993T>G mutation, with citrate and aconitate among the most significant metabolic rescues. Of note, production of citrate from oxaloacetate and acetyl-CoA is the first committed step of the tricarboxylic acid (TCA) cycle. This reaction requires intact mitochondrial function and favorable NAD+/NADH ratio (44,45). Therefore, the gradual increase in citrate upon correction of m.8993T>G mutation can be interpreted as a gradual restoration of TCA cycle activity underpinned by rescue of mitochondrial function. However, we cannot exclude that restoration of citrate and aconitate levels in N10 can be explained by additional metabolic changes, beyond increased citrate synthesis. Overall, these results are compatible with the notion that near-complete heteroplasmic shifts, obtained through mtZFN treatment, not only correct the m.8993T>G mutation, but also the bioenergetic and metabolic defects observed in cells bearing high levels of mutated mtDNA.

Significant variation across a range of physiological measures is often detected between cell lines of variable origin bearing the same mtDNA mutations, or between cybrid clones from the same genetic source bearing the same mtDNA mutation. It is apparent to most of the field that inherent variation between cybrid clones, which would otherwise be considered ‘isogenic’, can lead to uninterpretable results. To evidence this, we have measured respiration in an independently selected cybrid clone bearing 100% m.8993T>G mtDNA (N100). N100 respired at levels significantly higher than N80, N45 and N10, with commensurate elevation in energy charge state (Supplementary Figure S10), and was therefore deemed an inappropriate control in these studies. We are inclined to suggest that the population of cells generated using mtZFN and FACS-based selection, where short-term expression of a mitochondrially targeted nuclease leads to permanent changes in mtDNA heteroplasmy, constitutes a more representative ‘isogenic distribution’ of cells (rather than randomly-picked individual clones upon long-term selection) (46) from which the genuine physiological impact of mtDNA mutations can be teased. Therefore, rather than sounding the death knell for cybrid cells, perhaps similar approaches to those described in this work could be of benefit to groups studying cellular physiology using cybrid models in the future.

Various approaches to shifting mtDNA heteroplasmy, beyond those discussed in the body of this paper, have been reported in the recent past. These include upregulation of autophagy through over-expression of the ubiquitin ligase Parkin (47) and restoration of wild-type mtDNA in human cells bearing mtDNA rearrangements through ectopic overexpression of a yeast recombination enzyme (48). There also exist reports describing the successful import of synthetic, mutation-specific anti-sense RNA to mammalian mitochondria for inhibition of pathogenic mtDNA replication (49,50). This work is based upon claims of harnessing a naturally occurring RNA import mechanism for controlled RNA delivery into mammalian mitochondria. A cryptic mitochondrial import system for nucleus-encoded tRNAs, observed in lower metazoans, has also been proposed to exist in human cells, however the details of the components involved remain ill-defined. Short synthetic RNAs, comprising two domains of the yeast cytosolic tRNA^Lys(CUU)^ (D-arm and F-hairpin), have been claimed to be delivered into human mitochondria using this cryptic mechanism (49). Further, published data from the same group claim that 5S cytosolic ribosomal RNA (5S rRNA) is localised to the mitochondrial matrix, forming part of the mitochondrial ribosome (51); it has also been proposed that fragments of 5S rRNA can function as a vector for delivering heterologous RNA into human mitochondria (52). Additionally, the RNA component of the nuclear RNase P ribozyme has been localised to human mitochondria, with its stem-loop reported to direct the import of exogenous transcripts into human mitochondria (53). However, reconciling reports of efficient RNA import into mammalian mitochondria with other available data is problematic. Firstly, mammalian mtDNA encodes a full complement of tRNAs necessary for its expression (1), so, in principle, there is no functional reason for import of nucleus-encoded tRNA into the organelle. Secondly, recent structures of the large mitoribosomal subunit have confirmed that cytosolic 5S rRNA is not a component of the human mitoribosome, with a mitochondrially encoded tRNA residing in its predicted location instead (54,55). Thirdly, it has been demonstrated that human mitochondrial RNase P does not require a trans-acting RNA for catalysis, but is composed of protein only (56). In accordance with the above points, any mitochondrial import of synthetic RNA molecules, based on a naturally occurring mechanism in human cells, would have to be, at best, sporadic and inefficient.

The use of CRISPR/Cas9 in human mitochondria has also been reported in conjunction with the previously mentioned inefficient (or deficient) and uncharacterised mammalian mitochondrial RNA import pathway (57). However, given the aforementioned constraints, until experiments demonstrating synthetic RNA import into human mitochondria can be widely and reliably reproduced by other researchers, mitochondrial CRISPR/Cas9 should remain a promising yet unfulfilled method (58).

In summary, the developments described in this work, demonstrating that heteroplasmy shifting activity of mitochondrially targeted nucleases can be greatly enhanced by minimising deleterious off-target effects, are likely to be of value to the field. Despite being unable to demonstrate this effect using mtTALENs (Supplementary Figure S6–S8), owing to an inability to generate TALE domains capable of selectively binding mutant mtDNA, it seems likely that an approach to achieve optimal dosage of mtTALENs specific to a mutant haplotype could be of similar benefit to that seen for mtZFNs. Future applications of mitochondrially targeted nuclease technologies appear, at least in the short-term, to be progressing most quickly towards therapy for diseases arising from heteroplasmic mutant mtDNA (59). The most likely, and current gold standard modality for delivering such a form of genetic therapy is the recombinant adeno-associated virus (AAV), and tissue-specific serotypes thereof. Although the AAV is generally considered to be a transient, non-integrating genetic medium, it has been shown in animal models that viral genomes persist, in an episomal state, for essentially the entire life-span of the lab animal, most reliably in post-mitotic or slowly-dividing tissues (60). In the context of the rapid, destructive effects of transient expression that any mitochondrially targeted nuclease can achieve within 24 h, according to our data, it seems likely that exerting a fine level of control over these designer enzymes will be key to achieving any successful therapeutic outcome.

## Supplementary Material

SUPPLEMENTARY DATA
