# Supplementary material for: Near-complete elimination of mutant mtDNA by iterative or dynamic dose-controlled treatment with mtZFNs
Source: Nucleic Acids Res. 2016 Jul 27;44(16):7804–16. doi: 10.1093/nar/gkw676 (PMC5027515; doi:10.1093/nar/gkw676)
Supplement: SUPPLEMENTARY DATA [file supp_44_16_7804__index.html]

Near-complete elimination of mutant mtDNA by iterative or dynamic dose-controlled treatment with mtZFNs — SUPPLEMENTARY DATA 

# Near-complete elimination of mutant mtDNA by iterative or dynamic dose-controlled treatment with mtZFNs

## SUPPLEMENTARY DATA

- SUPPLEMENTARY DATA
