# Supplementary material for: Near-complete elimination of mutant mtDNA by iterative or dynamic dose-controlled treatment with mtZFNs
Source: Nucleic Acids Res. 2016 Jul 27;44(16):7804–16. doi: 10.1093/nar/gkw676 (PMC5027515; doi:10.1093/nar/gkw676)
Supplement: SUPPLEMENTARY DATA [file supp_gkw676_nar-00170-h-2016-File006.pdf]

Near-complete elimination of mutant mtDNA by iterative or dynamic dose-controlled treatment with mtZFNs

SUPPLEMENTARY DATA

Figure S1 – Ultra-deep sequencing of RFLP PCR amplicons used for m.8993T>G heteroplasmy measurements
Figure S2 – Expression of transiently transfected mtZFN over time in FACS enriched cells
Figure S3 – Quantification of m.8993T>G heteroplasmy in cells transiently transfected with mtZFNs
Figure S4 – Effects of utZFN expression dosage on mtDNA copy number in N80 cells
Figure S5 – A mitochondrially targeted restriction endonuclease, mt-*Xma*I
Figure S6 – Architecture and mitochondrial localisation of mtTALEN
Figure S7 – Effect of short-term mtTALEN expression on mtDNA heteroplasmy
Figure S8 – Effect of variable mtTALEN dosage on mtDNA heteroplasmy
Figure S9 – LC-MS-based metabolomics of m.8993T>G cells
Figure S10 – Oxygen consumption rate (OCR) and energy charge state analysis of N100 vs N80 lineage cells

SUPPLEMENTARY REFERENCES

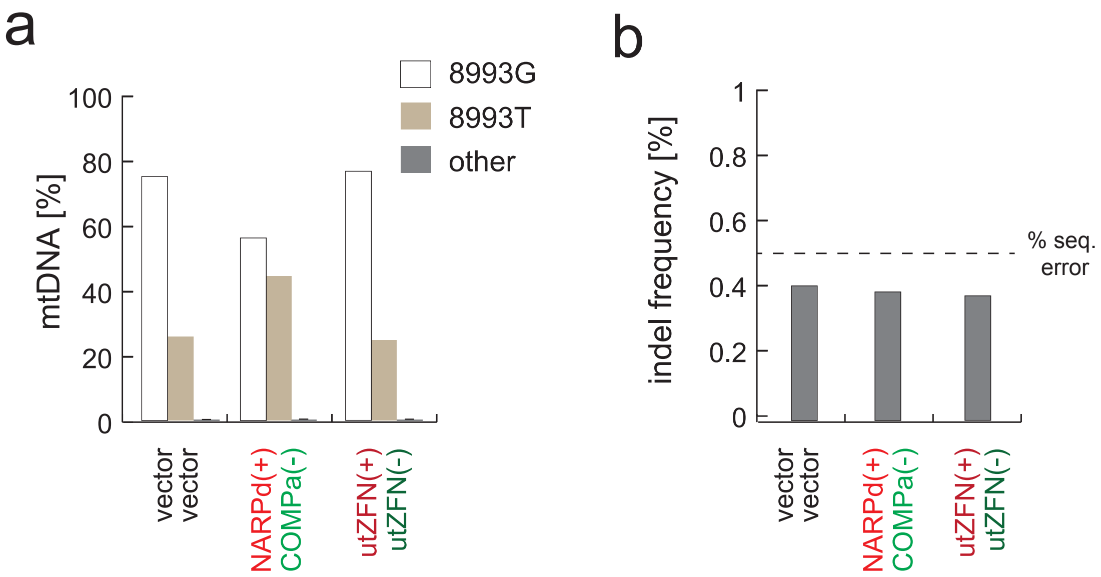

Figure S1. Ultra-deep sequencing of RFLP PCR amplicons used for of m.8993T>G heteroplasmy measurements. (**A**) Graphical representation of data acquired for samples of N80 cells transfected with indicated constructs, sorted by FACS at 24 hours and harvested at 28 days post-transfection. Heteroplasmy values were calculated, using VarScan, from at least 2x10E6 reads per sample. These data essentially recapitulate those obtained by *Sma*I RFLP analysis of the very same amplicon (**Figure 1D**, main text). No other mtDNA haplotypes were detected above noise. (**B**) Graphical representation of indel frequency as calculated by VarScan. A very low background level of indel activity is detected, below the likely sequencing noise threshold. There is no difference between conditions. Of note, the significant majority of indel detection was within two homopolymeric regions, indicating sequencing error rather than true indel occurrence.

These data confirm that LCH PCR and RFLP of nt.8339-9334 of mtDNA is an accurate measure of m.8993T>G heteroplasmy. Additionally, these results strongly suggest the absence of any classical NHEJ DNA double-strand break repair pathway functioning at a physiologically relevant level in human mitochondria.

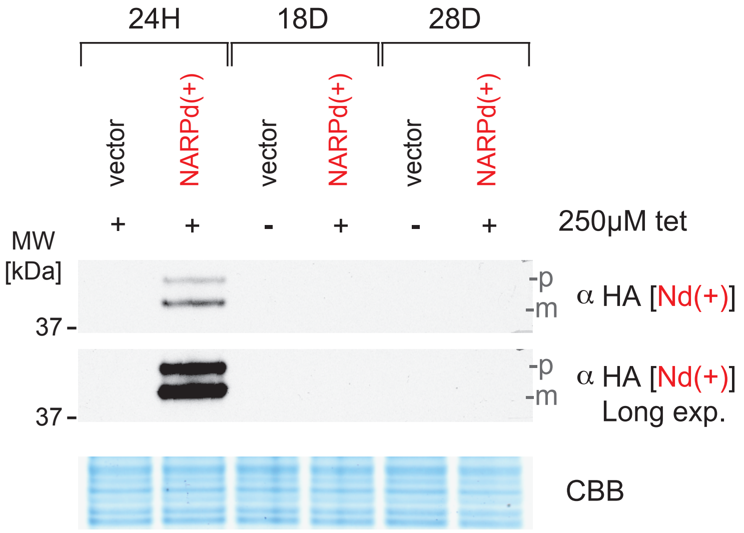

Figure S2. Expression of transiently transfected mtZFN over time in FACS enriched cells. Western blot analysis of total cell lysates from N80 cells transfected with NARPd(+)HHR or control vector, in the presence of 250 μM tetracycline. Cells were subjected to FACS at 24 hours (24H) post-transfection, and samples were collected at 24 hours (24H), 18 days (18D) and 28 days (28D) post-transfection. A section of Coomassie stained gel (CBB) is shown as a loading control. p - precursor isoform. m - mature isoform.

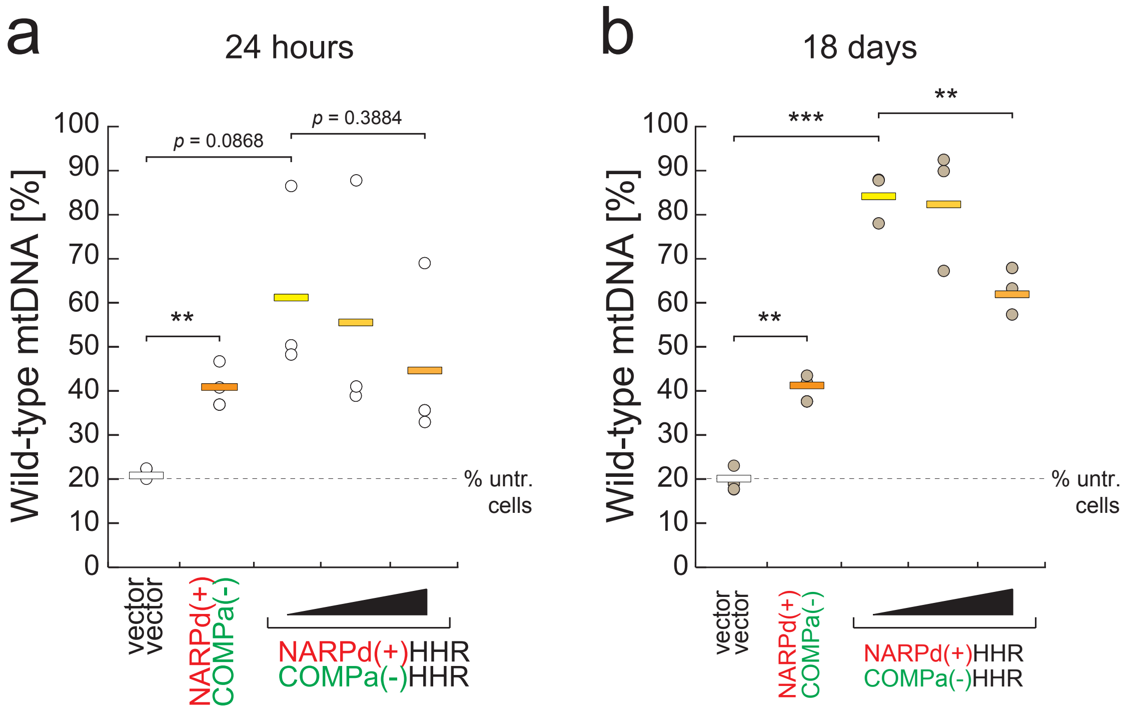

Figure S3. Quantification of m.8993T>G heteroplasmy in cells transiently transfected with mtZFNs (**A**) Collated data taken from several (n = 3) biological replicates. Data points shown are from samples measured at 24 hours post-transfection. Statistical analyses were conducted using a two-tailed student's *t*-test, P = 0.00457 (vector/mtZFN). "% untr. cells" indicates the baseline heteroplasmy of the N80 cell line used in these experiments. (**B**) Collated data taken from several (n = 3) biological replicates. Data points shown are from samples measured at 18 days post-transfection. Statistical analyses were conducted using a two-tailed Student's *t*-test, p = 0.00101 (vector/mtZFN), p = 0.00061 (vector/mtZFN-HHR, 0 μM), p = 0.00982 (mtZFN-HHR, 0 μM, mtZFN-HHR, 250 μM). "% untr. cells" indicates the baseline heteroplasmy of the N80 cell line used in these experiments.

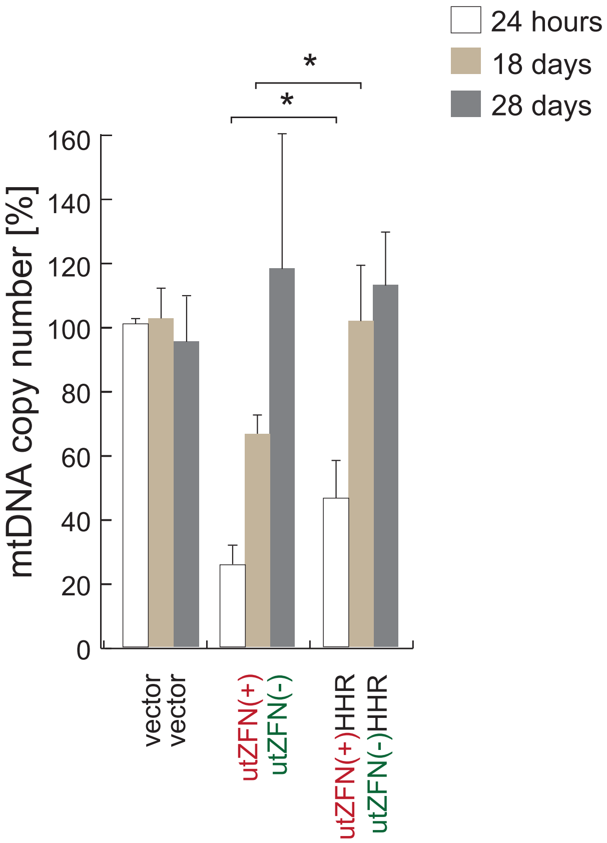

Figure S4. Effects of utZFN expression dosage on mtDNA copy number in FACS enriched N80 cells. Analysis of mtDNA copy number, performed by qPCR in quadruplicate on three independent biological repeats. utZFN(+/-)HHR transfected cells were cultured without tetracycline. Statistical analyses were conducted using a two-tailed Student's *t*-test: 24 hours (p = 0.01889, n = 3-8), 18 days (p = 0.02146, n = 3-8).

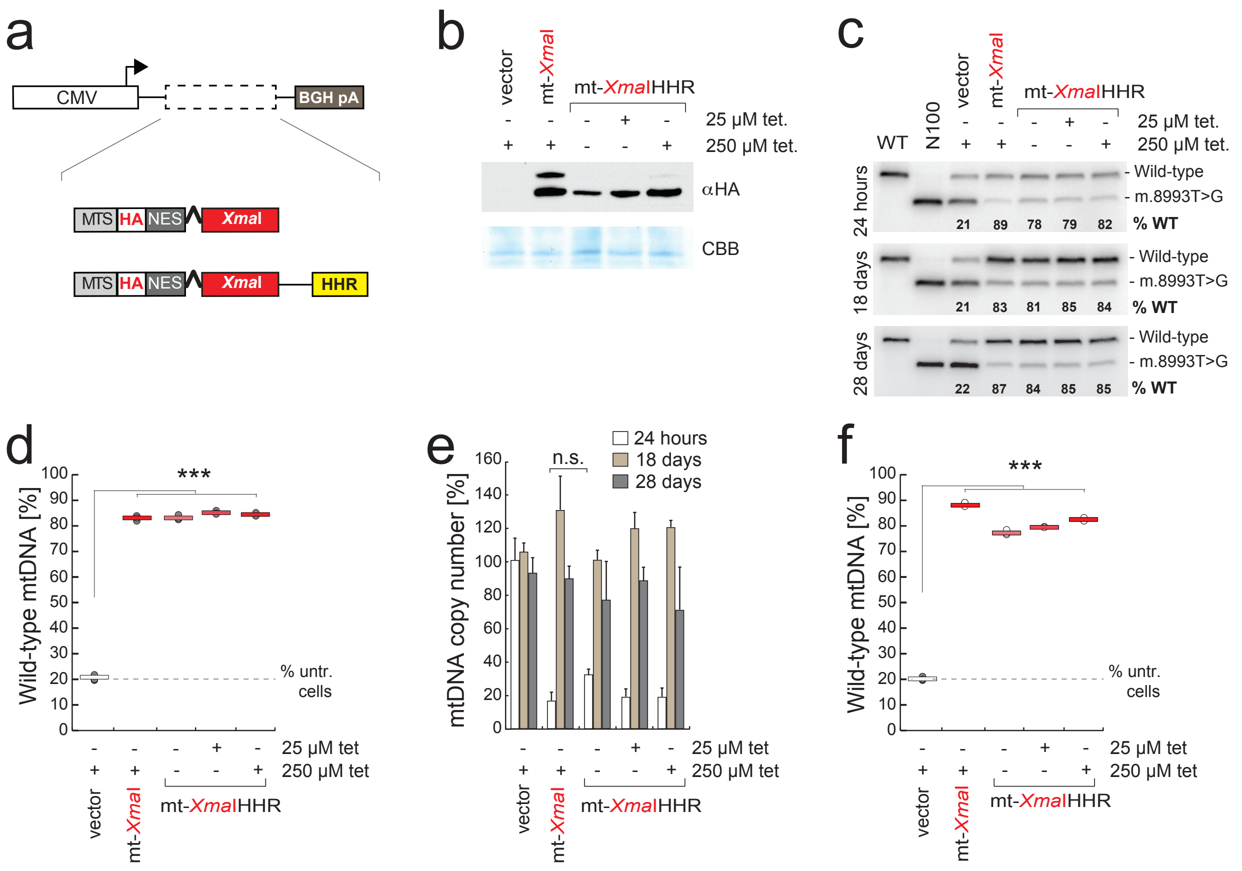

Figure S5. Effect of mt-*Xma*I expression on m.8893T>G heteroplasmy. (A) For the purpose of these experiments, the ORF of *Xma*I (optimised for mammalian expression, synthesized by GeneArt) was cloned immediately downstream of the MTS_HA_NES backbone used for mtZFN(+), displacing the DNA binding domain and *Fok*I domain, termed mt-*Xma*I. A further variant, incorporating the 3' hammerhead ribozyme (HHR) element, mt-*Xma*IHHR, was also produced. (**B**) Western blot analysis of total cell lysates from transiently transfected cells, acquired through FACS, supplemented with varying amounts of tetracycline in the media. A section of Coomassie stained gel (CBB) is shown as a loading control. (**C**) Last cycle hot PCR RFLP of mtDNA from cells transiently expressing various quantities of mt-*Xma*I in the presence of indicated quantities of tetracycline. Data from 24 hours, 18 days and 28 days post-transfection is shown. (**D**) Collated data taken from several (n = 3) technical replicates. Data points shown are from samples measured at 28 days post-transfection. Statistical analyses were conducted using a two-tailed Student's t-test, all conditions $p \leq 0.00002$. "% untr. cells." indicates the baseline heteroplasmy of the N80 cell line used in these experiments. (**E**) Analysis of mtDNA copy number, performed by qPCR in quadruplicate from samples tested in C. Error bars = 1 SD (**F**) Collated data taken from several (n = 3) technical replicates. Data points shown are from samples measured at 24 hours post-transfection. Statistical analyses were conducted using a two-tailed Student's t-test, all conditions $p \leq 0.00002$. "% untr. cells." indicates the baseline heteroplasmy of the N80 cell line used in these experiments.

These experiments demonstrate an alternate relationship between increased expression levels and heteroplasmy shifts to that demonstrated by mtZFN in experiments presented in Figure 3 of the main text. This is likely due to the capacity of the *Xma*I restriction endonuclease to reject a false substrate (defined as 1/6 bases of the recognition site being incorrect) at levels several orders of magnitude greater than a ZFN can likely claim [Withers & Dunbar, 1995]. Indeed, instead, we observe decreased heteroplasmy shifting activity when levels of mt-*Xma*I are reduced through HHR mediated mRNA degradation (Fig S2 E).

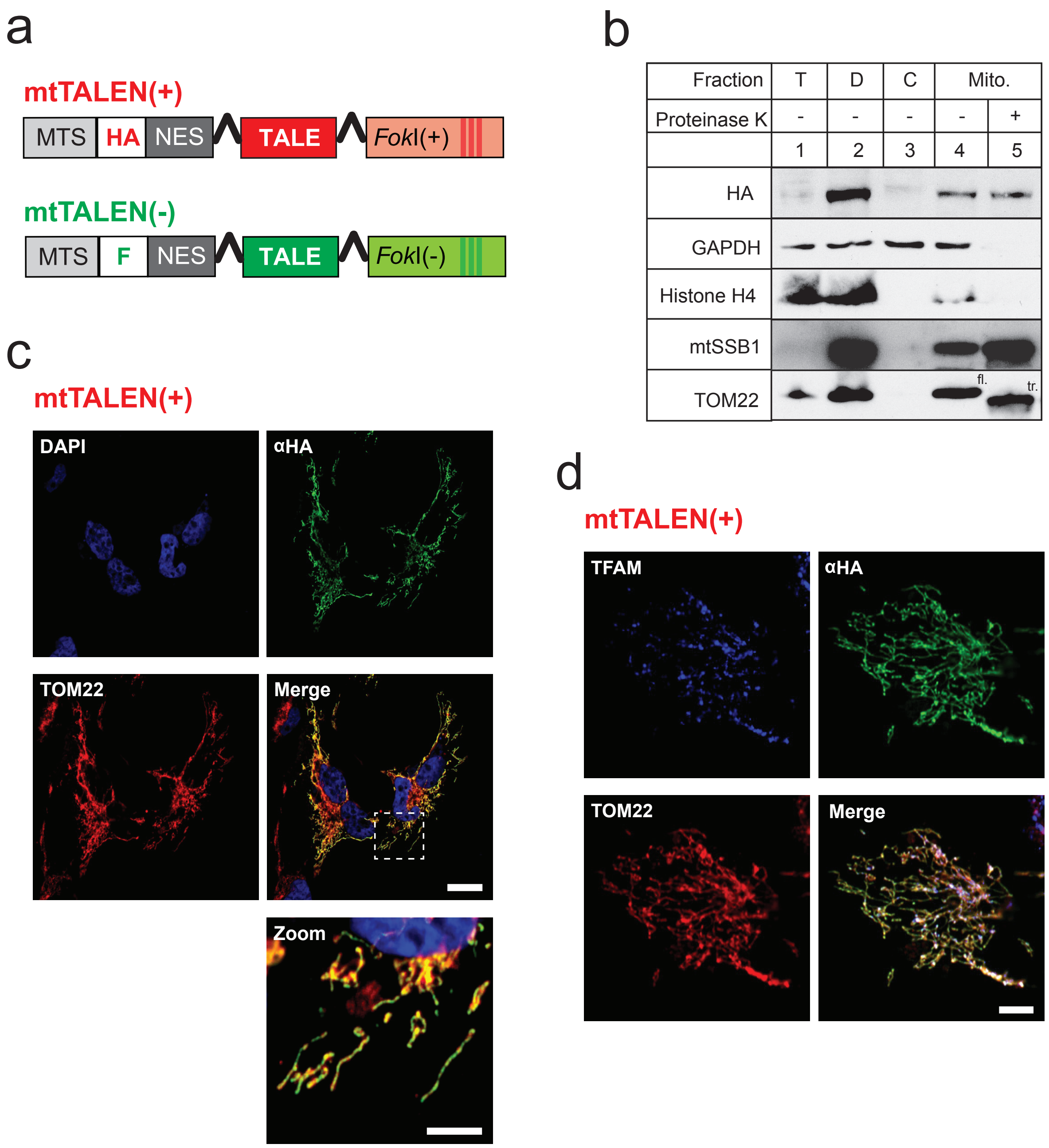

Figure S6. Architecture and mitochondrial localisation of mtTALEN. (**A**) Schematic of mtTALEN(+) and mtTALEN(-) monomers. All components, with the exception of the TALE domains, are identical to those used for mtZFN(+) and mtZFN(-) as previously reported. (**B**) Subcellular fractionation of HOS cells stably expressing mtTALEN(+) by mechanical disruption, differential centrifugation and protease treatment. mtTALEN(+) are found, by HA epitope immunodetection, to be significantly enriched in the mitochondrial fraction, and are spared from limited proteinase K treatment, indicating mitochondrial localisation. (**C**) Immunofluorescence analysis of HOS cells transiently transfected with mtTALEN(+). Nuclei were stained with DAPI, mitochondria were detected using anti-TOM22 and Alexafluor-594 conjugated antibodies, mtTALEN(+) were visualised using anti-HA and Alexafluor-488 conjugated antibodies. Images were digitally overlaid in the merge panel, clearly indicating mitochondrial localisation of mtTALEN(+). Scale bars: 10 µm. (**D**) Immunofluorescence analysis of HOS cells transiently transfected with mtTALEN(+). Mitochondrial nucleoids were visualised using anti-TFAM and Alexafluor-405 conjugated antibodies, mitochondria were detected using anti-TOM22 and Alexafluor-594 conjugated antibodies, mtTALEN(+) were visualised using anti-HA and Alexafluor-488 conjugated antibodies. Images were digitally overlaid in the merge panel, displaying clear co-localisation of mtTALEN(+) with both mitochondrial nucleoids and the mitochondrial network. Scale bar: 10 µm.

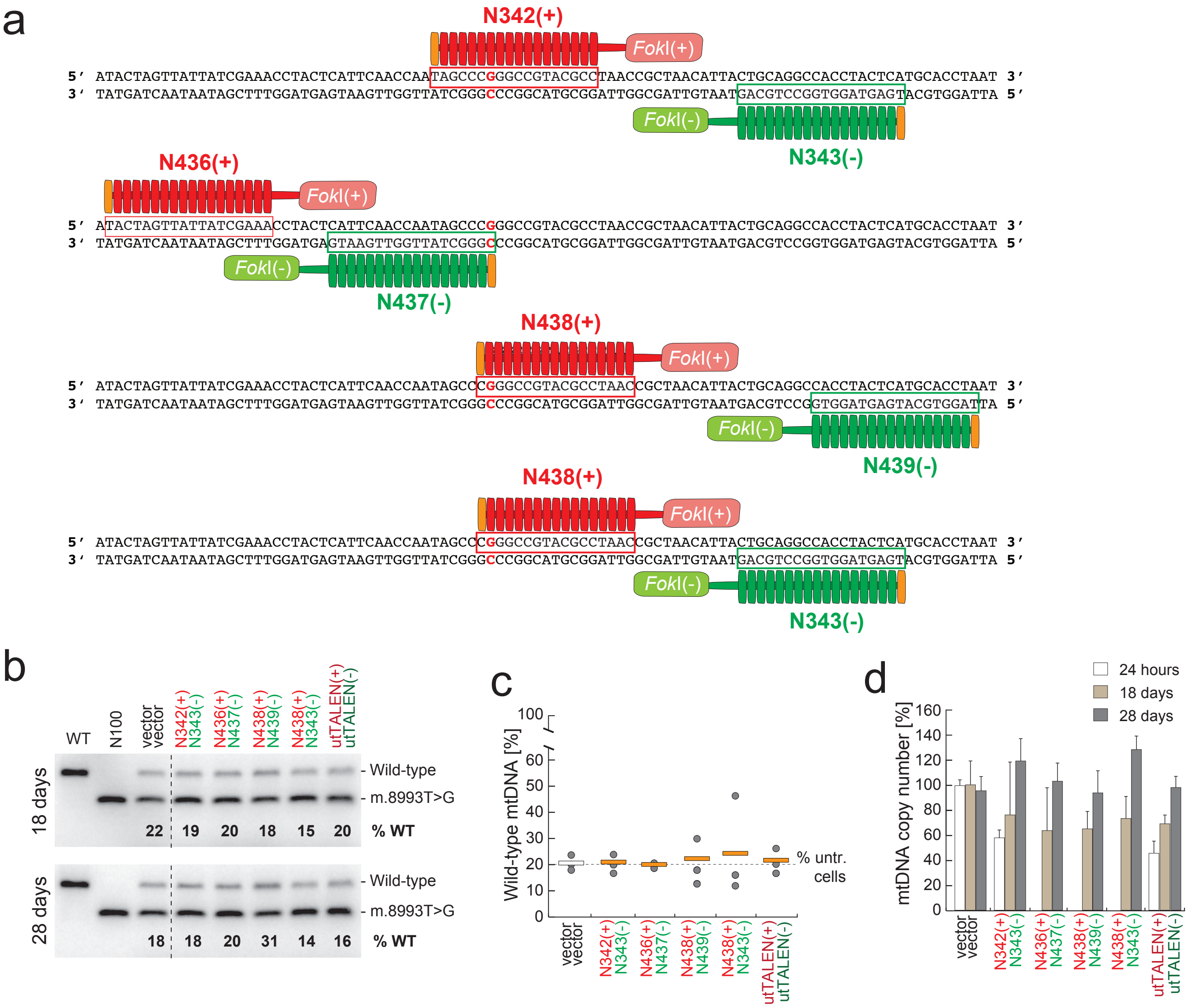

Figure S7. Assessing the effect of short-term high dose mtTALEN expression on m.8993T>G heteroplasmy. (A) Summary of TALE domains targeting m.8993T>G. Four distinct pairs of TALE domains were designed to target m.8993T>G mtDNA at optimal sites, considering implicit design constraints for effective TALE binding. Individual rectangles perpendicular to DNA sequence indicate individual TALE 33-35 aa DNA binding domains. Orange perpendicular rectangles indicate N-terminal cryptic repeat portion, essential for binding of TALE proteins. **(B)** Last cycle hot PCR RFLP analysis of mtDNA from FACS-enriched cells transiently expressing indicated mtTALEN construct pairs. **(C)** Quantification of RFLP heteroplasmy data from several biological replicates of FACS-enriched cells transiently transfected with mtZFN or control vectors. Data presented are from measurements taken at 18 days and 28 days post-transfection, as indicated. ”% untr. cells.” indicates the baseline heteroplasmy of the N80 cell line used in these experiments. (**D**) Analysis of mtDNA copy number, performed by qPCR in quadruplicate, from samples tested in C. Error bars = 1 S.D.

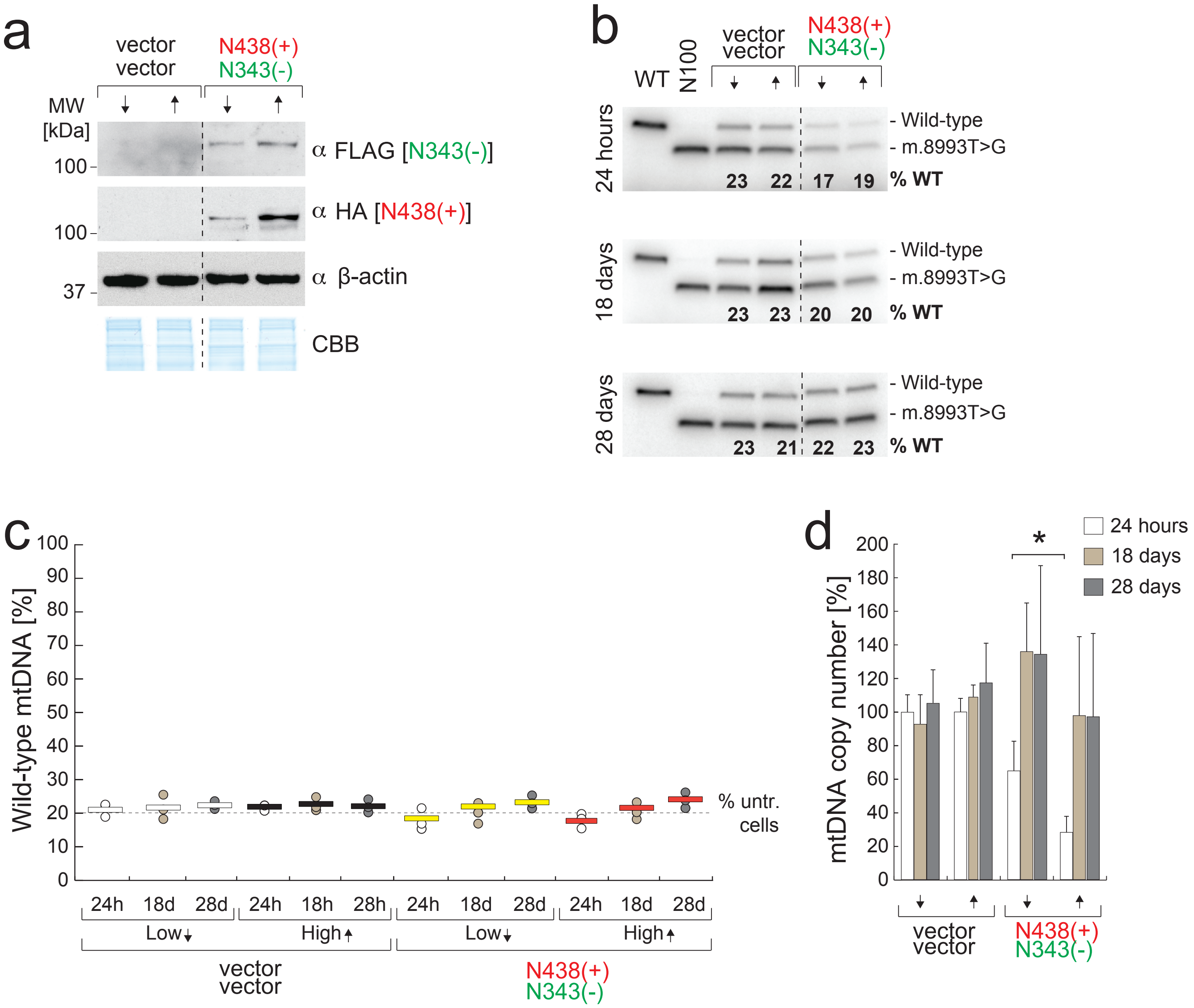

Figure S8. Assessing the effect of different dosage of mtTALENs on m.8993T>G heteroplasmy. **(A)** Western blot analysis of total cellular proteins by SDS-PAGE, probing for mtTALEN expression with antibodies to the HA (N438) or FLAG (N343) epitopes. β-actin and a section of Coomassie stained gel are shown as loading controls. (**B**) Last cycle hot PCR RFLP of mtDNA from cells transiently expressing 'high' or 'low' quantities of mtZFN or control vectors. (**C**) Quantification of collated heteroplasmy data from several biological replicates of transient mtTALEN or control vector expression. Data presented are from measurements made at 24 hours, 18 days and 28 days post-transfection, as indicated. "% untr. cells." indicates the baseline heteroplasmy of the N80 cell line used in these experiments. (**D**) Analysis of mtDNA copy number, performed by qPCR in quadruplicate, from samples tested in C. Statistical analyses were undertaken using a two-tailed Student's *t*-test, $p = 0.04709$. Error bars = 1 SD.

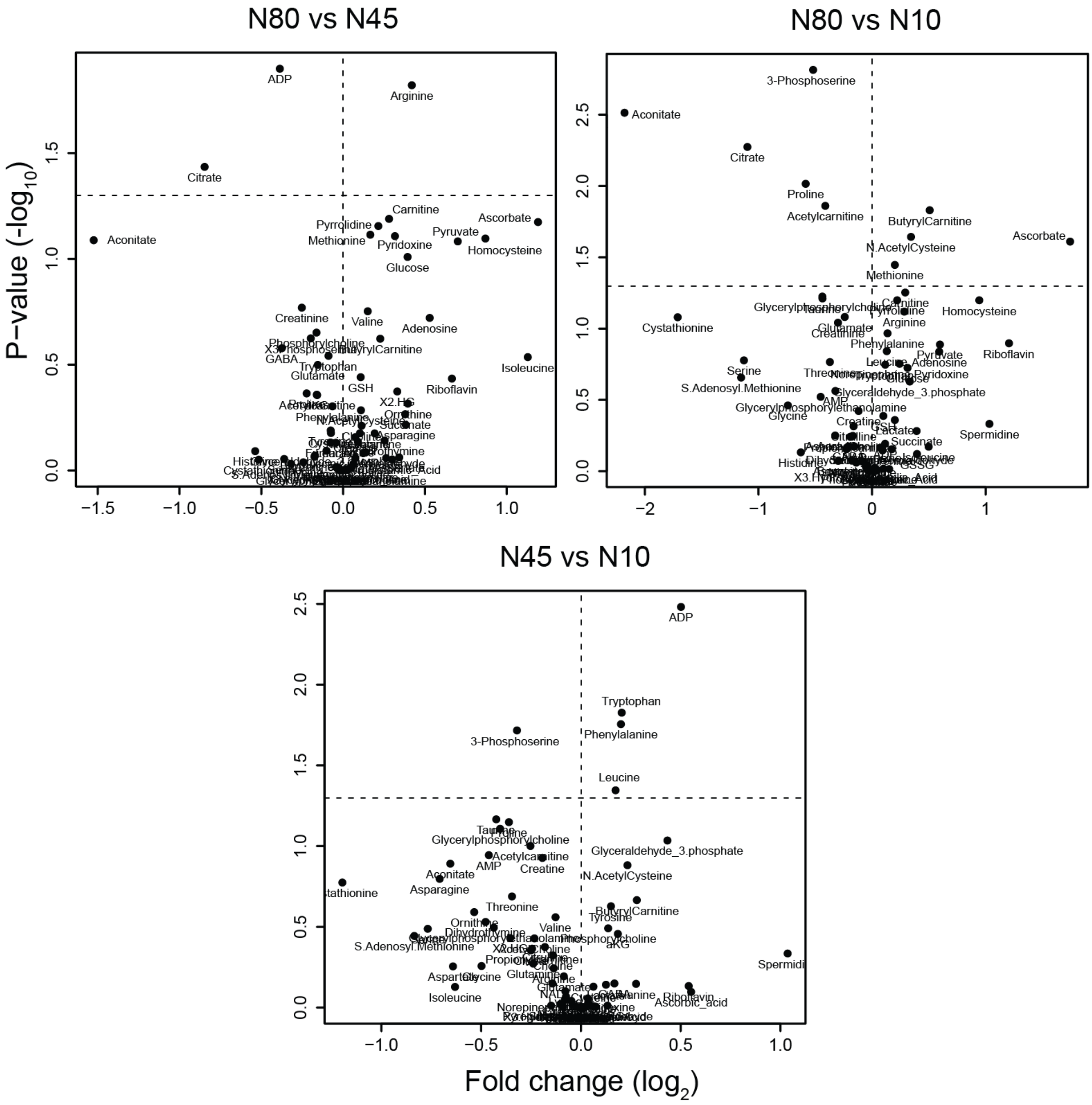

Figure S9. LC-MS-based metabolomics reveal substantial metabolic rewiring associated with correction of m.8993T>G mutation. Volcano plots reporting the $\log_2$ fold changes (X axis) and the $-\log_{10}$ p-values (Tukey) of 70 intracellular metabolites between indicated cell lines. Horizontal dashed lines indicate FDR levels of 5%.

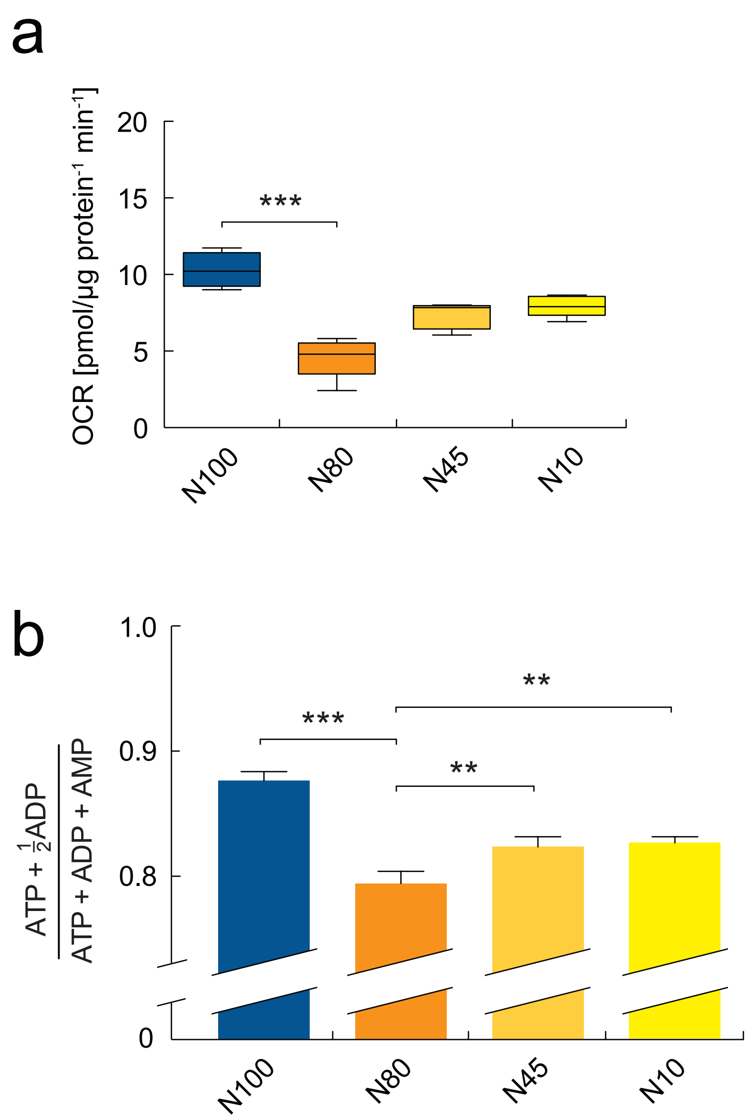

Figure S10. Oxygen consumption rate (OCR) and energy charge state analysis of N100 vs N80 lineage cells. (**A**) Basal OCR of 143B m.8993T>G cybrids as determined by microscale oxygraphy. Statistical analysis was conducted by two-tailed Student's *t* test (p = 0.000296, n = 4-8) (**B**) Energy charge state analysis of 143B m.8993T>G cybrids, as determined by LC-MS measures of adenylyl phosphate species. Statistical analyses were conducted by one-way ANOVA and Tukey's post-hoc test: N100 vs N80 (p <0.001, n = 3-4), N80 vs N45 (p < 0.01, n = 3), N80 vs N10 (p < 0.01, n = 3).

Issues of clonal diversity are unavoidable when producing cybrid cell lines. Such diversity often means that experiments comparing cybrid clones bearing various heteroplasmies of a single mtDNA variant are unreliable, giving spurious results that can be misinterpreted as genuine biochemical phenomena. From the experiments presented here, it is clear that N100, respiring at significantly higher levels than N80, with concomitantly elevated energy charge state, is an entirely inappropriate control for any metabolic or physiological measures.

SUPPLEMENTARY REFERENCES

Withers B.E. & Dunbar, J.C. 1995. DNA determinants in sequence-specific recognition by XmaI endonuclease. *J. Biol. Chem.* **23**: 3571-3577.
